# PReS13-SPK-1350: Autoimmune responses following vaccination in healthy populations

**DOI:** 10.1186/1546-0096-11-S2-I29

**Published:** 2013-12-05

**Authors:** N Toplak

**Affiliations:** 1 Department of Allergology, Rheumatology and Clinical Immunology, University Children's Hospital, University Medical Center, Ljubljana, Slovenia

## 

Vaccinations against infectious diseases are one of the major achievements in medicine in the last century and the most effective method for preventing infections. Concern about safety of vaccinations has been heightened by several reports of possible vaccine-induced autoimmune phenomena following various vaccinations. So far no study was able to show a casual connection between any vaccine and autoimmune syndrome.

Few studies were published showing that induction of autoantibodies following various vaccination is possible, but without clinical significance. In few cases antibodies after vaccination were elevated even 6 months after vaccination. Induction of autantibodies, mainly antiphospholipid antibodies, in selected apparently healthy individuals was reported after influenza, hepatitis B and hepatitis A vaccination.

Autoimmune manifestations reported have been only temporally related to the respective vaccine. Guillian Barre syndrome was described following vaccination against influenza and few other vacines, multiple sclerosis and arthritis were mainly reported after hepatitis B vaccination. There are some evidence to suggest a connection of reactive arthritis and rubella vaccine. Dermatomyositis was described following hepatitis B, tuberculosis, influenza and tetanus vaccinations. Recently a new syndrome Autoimmune-Autoinflammatory syndrome induced by adjuvants-ASIA has been described.

Highlights of lecture:

- autoimmune adverse events following vaccinations is possible in selected individuals but the risk of autoimmune disease after vaccination is, comparing to advantages of vaccination, negligible.

- induction of autoantibodies in selected individuals after vaccination has no clinical significance.

- new generation vaccine, mainly oriented on finding safer and effective adjuvants, are needed. At present few effective adjuvants are considered safe for use in humans.

## Disclosure of interest

None declared.

